# Mass Cytometry Analysis Reveals the Landscape and Dynamics of CD32a^+^ CD4^+^ T Cells From Early HIV Infection to Effective cART

**DOI:** 10.3389/fimmu.2018.01217

**Published:** 2018-06-04

**Authors:** Sixtine Coindre, Nicolas Tchitchek, Lamine Alaoui, Bruno Vaslin, Christine Bourgeois, Cecile Goujard, Veronique Avettand-Fenoel, Camille Lecuroux, Pierre Bruhns, Roger Le Grand, Anne-Sophie Beignon, Olivier Lambotte, Benoit Favier, The ANRS CO6 PRIMO Cohort

**Affiliations:** ^1^CEA-Université Paris Sud 11-INSERM U1184, Immunology of Viral Infections and Autoimmune Diseases (IMVA), IDMIT Department, IBFJ, DRF, Fontenay-aux-Roses, France; ^2^Assistance Publique-Hôpitaux de Paris, Service de Médecine Interne et Immunologie Clinique, Groupe Hospitalier Universitaire Paris Sud, Hôpital Bicêtre, Le Kremlin-Bicêtre, France; ^3^Paris Descartes University, EA 7327, Sorbonne Paris Cité, APHP, Necker Hospital, Virology Department, Paris, France; ^4^Institut Pasteur, Department of Immunology, Unit of Antibodies in Therapy and Pathology, Paris, France; ^5^INSERM, U1222, Paris, France

**Keywords:** CD4^+^ T-lymphocytes, CD32a, mass cytometry CyTOF, primary HIV infection, combination antiretroviral therapy

## Abstract

CD32a has been proposed as a specific marker of latently HIV-infected CD4^+^ T cells. However, CD32a was recently found to be expressed on CD4^+^ T cells of healthy donors, leading to controversy on the relevance of this marker in HIV persistence. Here, we used mass cytometry to characterize the landscape and variation in the abundance of CD32a^+^ CD4^+^ T cells during HIV infection. To this end, we analyzed CD32a^+^ CD4^+^ T cells in primary HIV infection before and after effective combination antiretroviral therapy (cART) and in healthy donors. We found that CD32a^+^ CD4^+^ T cells include heterogeneous subsets that are differentially affected by HIV infection. Our analysis revealed that naive (_N_), central memory (_CM_), and effector/memory (_Eff/Mem_) CD32a^+^ CD4^+^ T-cell clusters that co-express LILRA2- and CD64-activating receptors were more abundant in primary HIV infection and cART stages. Conversely, LILRA2^−^ CD32a^+^ CD4^+^ T-cell clusters of either the T_N_, T_CM_, or T_Eff/Mem_ phenotype were more abundant in healthy individuals. Finally, an activated CD32a^+^ CD4^+^ T_Eff/Mem_ cell cluster co-expressing LILRA2, CD57, and NKG2C was more abundant in all HIV stages, particularly during primary HIV infection. Overall, our data show that multiple abundance modifications of CD32a^+^ CD4^+^ T-cell subsets occur in the early phase of HIV infection, and some of which are conserved after effective cART. Our study brings a better comprehension of the relationship between CD32a expression and CD4^+^ T cells during HIV infection.

## Introduction

Effective combination antiretroviral therapy (cART) leads to the control of HIV replication and disease progression in HIV-infected patients (1, 2). Yet, a reservoir of latently infected CD4^+^ T cells persists despite effective cART and can rapidly re-establish high viremia following treatment interruption (3–6). Hence, characterization of latently infected CD4^+^ T cells is required for the design of therapeutic strategies to target HIV reservoirs. In this respect, a recent study showed that the Fc receptor CD32a (also known as FcγRIIa) could be a critical marker for a substantial portion of the latently HIV-infected CD4^+^ T cells that harbor replication-competent proviruses (7). However, CD32a expression was also reported on CD4^+^ T cells displaying HIV replication and on CD4^+^ T cells from healthy donors (8–11), leading to controversy over the relevance of this marker for the characterization of latent HIV reservoirs. Moreover, CD4^+^ T cells include naive, effector, and memory populations that are differentially expanded and infected by HIV, in particular during primary infection (12, 13). Thus, evaluating the heterogeneity of CD32a^+^ CD4^+^ T cells in early HIV infection, before and after effective cART, could be helpful to better characterize the relationship between CD32a expression and HIV infection.

Here, we carried out a pan-leukocyte analysis of CD32a expression by mass cytometry to assess CD32a^+^ CD4^+^ T-cell diversity in primary HIV-infected patients longitudinally collected before and after 12 months of effective cART. We then compared differences in the abundance of the identified CD32a^+^ CD4^+^ T-cell subpopulations among all HIV^+^ stages and under healthy conditions. CD32a is generally found on myeloid cells but also appears to be specifically found on a subpopulation of CD4^+^ T cells associated with HIV persistence (7). Thus, our pan-leukocyte analysis included lymphoid and myeloid markers that could help to better characterize the phenotype and heterogeneity of CD32a^+^ CD4^+^ T cells. In this respect, we explored the expression of inhibitory and activating leukocyte immunoglobulin-like receptors (LILRs) that are distantly related to FcRs (14). LILRs are mainly distributed on myeloid cells, but some members are also found on T-cell subsets, similar to FcRs (15–18). Moreover, LILRs can directly regulate the activity of CD32a and CD64 (19–22) and play an important role in the dysregulation of immune responses against HIV (23–26).

Previous studies analyzed CD32 expression with only a pan-CD32 antibody. We thus also included antibodies that discriminate CD32a from CD32b to unambiguously identify CD32a^+^ CD4^+^ T cells. We also investigated the expression of CD16 and CD64, belonging to the FcR family, within the CD32a^+^ CD4^+^ T-cell population.

Here, we show that primary HIV and cART stages are associated with a higher proportion of CD32a^+^ CD4^+^ T cells, co-expressing LILRA2 and CD64, among naïve (T_N_), central memory (T_CM_), and effector/memory (T_Eff/Mem_) subpopulations. In addition, we found that a subset of activated T_Eff/Mem_ CD32a^+^ LILRA2^+^ CD57^+^ NKG2C^+^ cells was more abundant in all HIV stages and positively correlated with HIV DNA levels.

Altogether, our results unravel the diversity of CD32a^+^ CD4^+^ T cells and the various phenotypic changes that occur during early immune responses against HIV and after effective cART.

## Results

### Mass Cytometry Analysis Reveals the Diversity of CD32a^+^ CD4^+^ T-Cell Populations Among HIV-Infected Patients and Healthy Donors

We characterized the heterogeneity of CD32a^+^ CD4^+^ T-cell populations in primary HIV-infected patients, before and after effective cART, as well as in healthy donors, using a pan-leukocyte mass cytometry panel of 35 markers. For this purpose, a first group of six patients were longitudinally sampled at the time of diagnosis, in the primary phase of HIV infection (primary HIV), and after 12 months of successful cART (HIV cART). Primary HIV infection referred to blood samples collected between 18 and 30 days after HIV infection (Fiebig stage III/IV) with a high-level plasma viral load (number of copies/ml > 200,000) including parameters described by Krastinova et al. (27).

**Figure 1:**
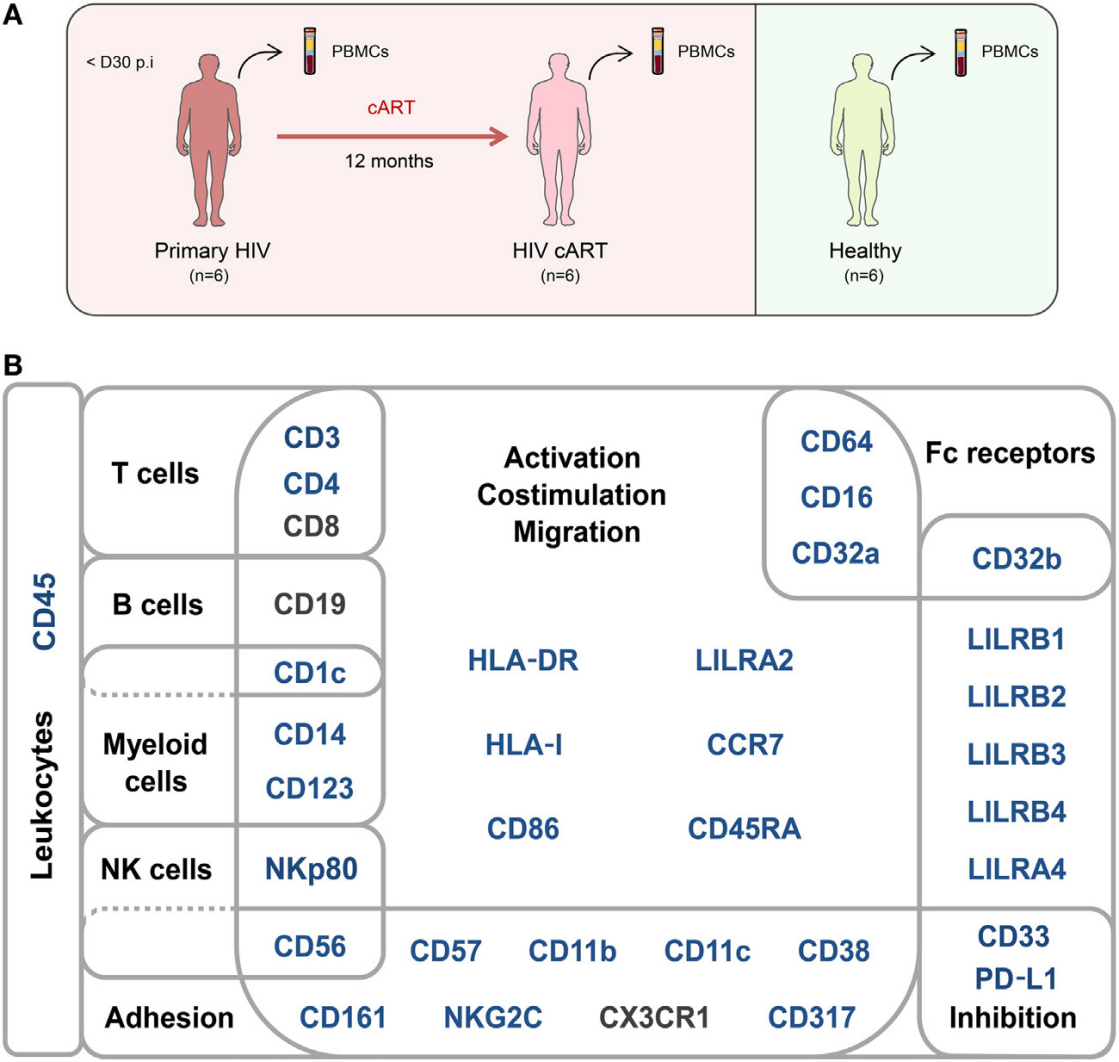
Study design and mass cytometry panel. **(A)** Schematic representation of the study design. Two groups of individuals were studied. The first group corresponds to HIV-infected patients collected during primary HIV infection, diagnosed within 30 days post-infection (primary HIV), and after 12 months of combination antiretroviral therapy (HIV cART). The second group corresponds to healthy donors (healthy). Blood samples were collected from the two groups of individuals and PBMCs isolated for mass cytometry analysis. **(B)** Pan-leukocyte mass cytometry panel, including 35 parameters, used to characterize CD32a^+^ CD4^+^ T-cell diversity in leukocytes from PBMC samples. Markers used for Spanning-tree Progression Analysis of Density-normalized Events (SPADE) clusterization of CD4^+^ T cells are indicated in blue. Non-SPADE clustering markers are indicated in gray.

A second group of individuals was comprised of six healthy HIV-negative donors (healthy) (Figure 1A). Clinical characteristics of these groups are shown in Table 1. The pan-leukocyte mass cytometry panel included markers for lymphoid and myeloid cells, migration and adhesion markers, and activating and inhibitory immunoreceptors (Figure 1B). CD32 includes highly homologous isoforms (15). We thus used monoclonal antibodies that specifically recognize either CD32a (clone IV.3) or CD32b (clone 2B6) to avoid misinterpretation of the data (Table S1 in Supplementary Material). The specificity of these antibodies was confirmed by the detection of CD32a, but not CD32b, on monocytes and CD32b on only B cells (Figure S1 in Supplementary Material), in agreement with previous studies (15). Thus, our results unambiguously reflect CD4^+^ T cells expressing CD32a protein on their surface.

**Table 1:** Summary of patient and subject clinical parameters.

	Primary HIV (*n* = 6)	→	HIV cART (*n* = 6)	Healthy (*n* = 6)
Age Median (min–max), in years	34 (24–47)		35 (25–48)	35 (25–45)

Gender	M		M	M

Days since HIV-1 diagnosis Median (min–max), in days	28 (18–29)		361 (290–372)	N/A

Treatment	None		cART	None

HIV DNA Median (min–max), in log_10_ copies/10^6^ PBMCs	3.55 (2.90–3.90)		2.21 (1.39–2.74)	N/A

HIV RNA load Median (min–max), in log_10_ copies/ml of plasma	6.67 (5.47–7.26)		1 (1–1.63)	N/A

CD4^+^ T-cell count Median (min–max), in 10^3^ cell/μl of blood	470 (258–669)		843 (570–1,247)	856 (634–1,412)

*Twelve Caucasian men were involved in this study to constitute two groups of individuals. These groups are composed of six primary HIV-infected patients before treatment (primary HIV) and after 12 months of combination antiretroviral therapy (HIV cART) and six healthy subjects (healthy). The gender and median patient age, days since HIV-1 diagnosis, total DNA level, RNA viral load, and CD4^+^ T-cell count are indicated for each group*.

*cART, combination antiretroviral therapy; N/A, not applicable; M, male*.

We applied a gating strategy to select the whole CD4^+^ T-cell population (CD19^−^, CD3^+^, CD8^−^, and CD4^+^) from total leukocytes (CD45^+^) (Figure S2 in Supplementary Material). We next characterized the simultaneous expression of the markers from our panel on total CD4^+^ T cells by performing a Spanning-tree Progression Analysis of Density-normalized Events (SPADE) (28). The SPADE algorithm aims to identify cell clusters with similar expression of selected markers, regardless of the sample cell origin (29). We benchmarked a set of parameters (as described in section “Materials and Methods”) to optimize the SPADE analysis. We obtained 322 CD4^+^ T-cell clusters from all cell samples, after excluding a few contaminant myeloid cell clusters, and generated a heatmap representing their respective relative marker expression (Figure S3 in Supplementary Material). The range of expression for each marker (5th to 95th percentiles of expression) is represented using a gradient color scale ranging from white (not expressed) to dark red (highly expressed) (Figure S4 in Supplementary Material). Some CD4^+^ T-cell clusters exhibited CD32a at their surface at various levels, as previously reported (7).

**Figure 2:**
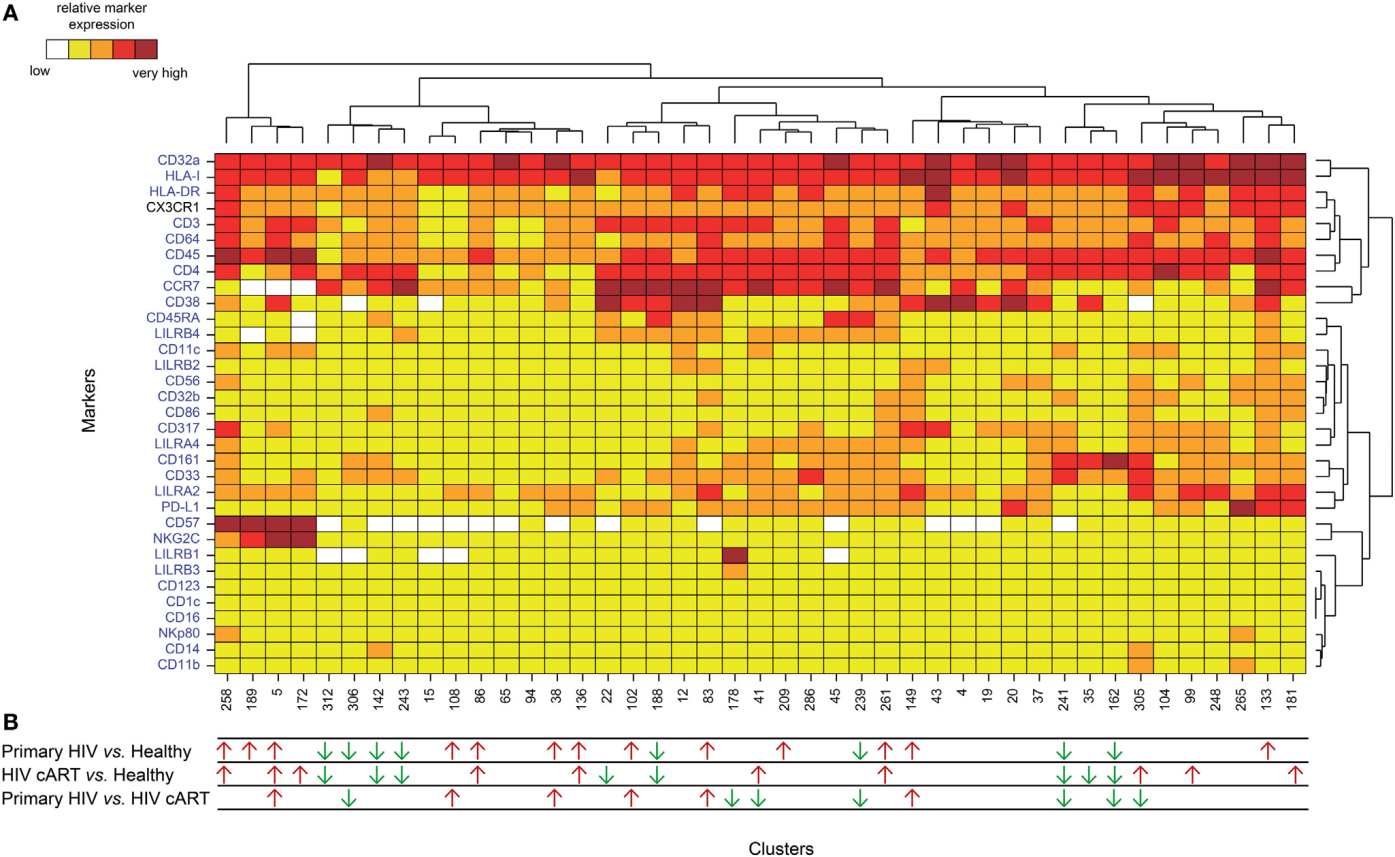
Phenotypic landscape and variation in cell abundance of CD32a^+^ CD4^+^ T-cell clusters from HIV-infected and healthy donor samples. **(A)** Heatmap showing relative marker expression for CD32a^+^ CD4^+^ T-cell clusters. The mean of the median expression of each marker was determined and classified using a five-tiered color scale, from white (not expressed) to dark red (highly expressed), according to their range of expression (5th to 95th percentile) throughout the dataset. Clustering markers are shown in blue. Hierarchical clustering of both the cell clusters and clustering markers were performed and represented using dendrograms. **(B)** Chart summarizing the clusters showing significantly different cell abundances between the biological conditions [differentially abundant clusters (DACs)]. For each comparison of corresponding DACs, red arrows indicate an increase in the abundance of the cell cluster, whereas green arrows indicate a decrease in abundance, for the left condition relative to that on the right.

HIV latency was proposed to be associated with increased expression of CD32a (7). We thus focused on CD4^+^ T-cell clusters displaying high levels of CD32a (CD32a^+^), indicated on the heatmap in red and dark red (Figures S3 and S4 in Supplementary Material), and generated a second heatmap representing only CD32a^+^ CD4^+^ T-cell clusters (Figure 2A). The total number of cells (from all conditions) associated with each CD32a^+^ CD4^+^ T-cell cluster was quantified (Figure S5 in Supplementary Material). Characterization of these clusters revealed heterogeneous phenotypes within CD32a^+^ CD4^+^ T cells that were associated with naive (T_N_: CCR7^+^ CD45RA^+^), central memory (T_CM_: CCR7^+^ CD45RA^−^), and effector/memory (T_Eff/Mem_: CCR7^−^ CD45RA^−^) subsets (Figure 2A). Further analysis showed that among CD32a^+^ CD4^+^ T-cell clusters, HIV^+^ patients under cART displayed a significantly higher proportion of T_CM_ than T_N_ (*p* = 0.016) (Figure S6 in Supplementary Material). These subsets of CD32a^+^ CD4^+^ T_CM_ and T_N_ represented an average of 5.25 and 1.01% of HIV cART CD4^+^ T cells, respectively. CD32a was also expressed on CD4^+^ T cells from healthy donors (Figure S6 in Supplementary Material). In healthy donors, the proportion of CD32a^+^ CD4^+^ T cells was significantly higher among T_CM_ than T_N_ (*p* = 0.034), similar to HIV cART patients, representing an average of 3.37 and 1.20% of CD4^+^ T cells, respectively (Figure S6 in Supplementary Material). The percentages of CD32a^+^ CD4^+^ T_N_, T_CM_, and T_Eff/Mem_ also varied in primary HIV^+^ patients, but the differences were not statistically significant.

These results show that CD32a^+^ CD4^+^ T cells are present in HIV^+^ patients, as well as healthy donors, and are contained within heterogeneous populations showing naive, central memory, or effector/memory phenotypes that may be differentially affected by HIV infection.

### Clusters of LILRA2^+^ CD32a^+^ CD4^+^ T Cells Are More Abundant in HIV-Primary Infection and After Effective cART

We determined whether HIV infection was associated with specific CD32a^+^ CD4^+^ T-cell clusters by characterizing the differentially abundant clusters (DACs) between two conditions, including primary HIV vs. healthy donors, HIV cART vs. healthy donors, and primary HIV vs. HIV cART (Figure 2B). DACs were identified based on their cell abundance relative to that of CD32a^+^ CD4^+^ T cells. DACs showing a significantly (*p* < 0.05) higher cell proportion in each HIV^+^ stage (primary HIV and cART) vs. healthy donors (red arrows) were selected for further analysis.

**Figure 3:**
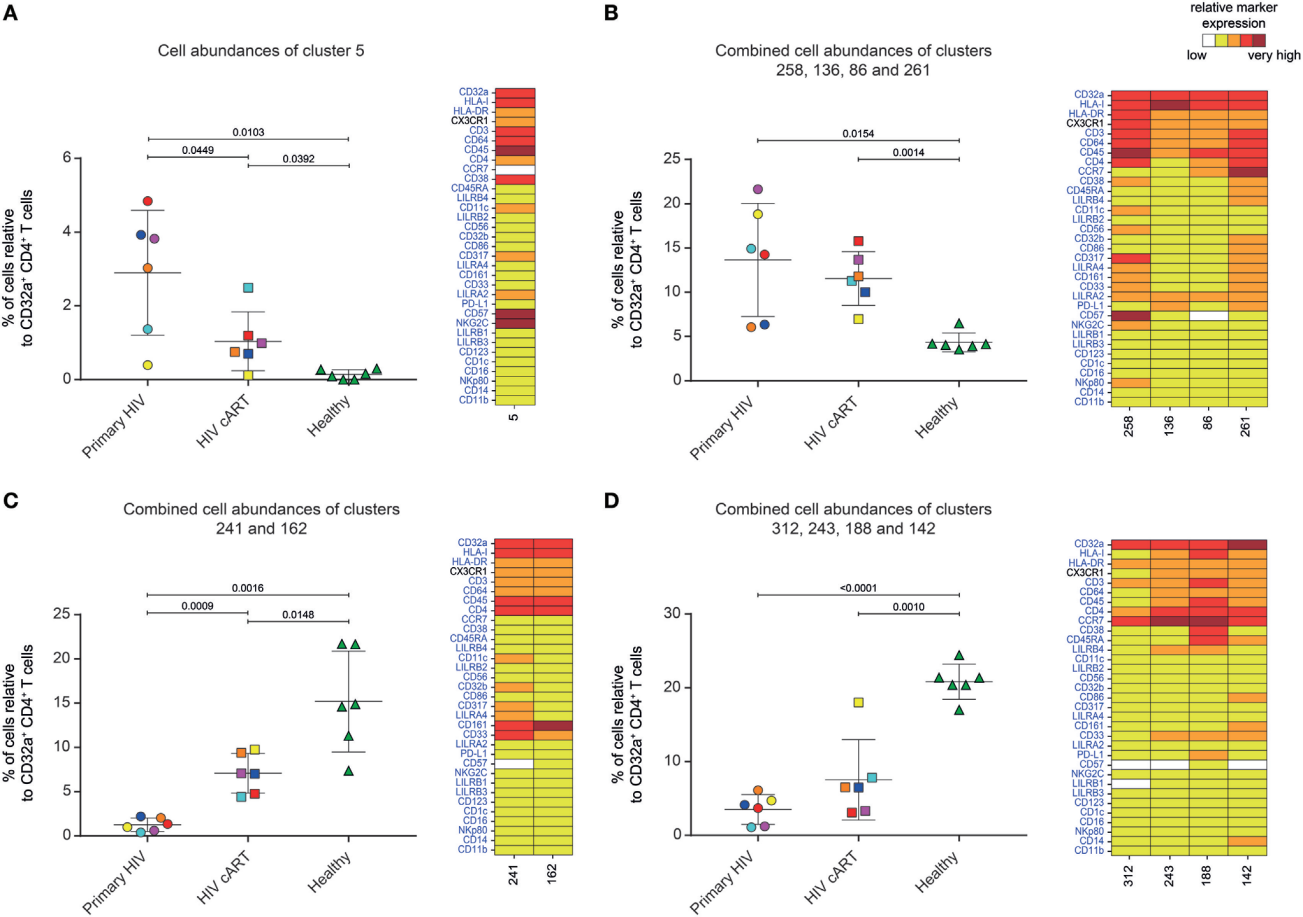
Characterization of CD32a^+^ CD4^+^ T-cell clusters showing significant differences in cell abundance for HIV^+^ conditions. **(A)** Graph showing the cell abundance of the cluster #5 relative to CD32a^+^ CD4^+^ T cells for all samples, and heatmap representation illustrating the phenotype of this cluster. Cluster #5 was significantly more abundant under primary HIV (circle) and HIV cART (square) than healthy (triangle) conditions. This cluster was also more abundant under primary HIV than HIV cART conditions. **(B)** Graph showing the cell abundance of the clusters #258, #136, #86, and #261 relative to CD32a^+^ CD4^+^ T cells for all samples, and heatmap representation of the phenotype of these clusters. These clusters were significantly more abundant under primary HIV and HIV cART than healthy conditions. **(C)** Graph showing the cell abundance of the clusters #241 and #162 relative to CD32a^+^ CD4^+^ T cells for all samples, and heatmap representation showing the phenotype of these clusters. Clusters #241 and #162 were significantly less abundant under primary HIV and HIV cART than healthy conditions. These clusters were also less abundant under primary HIV than HIV cART conditions. **(D)** Graph showing the cell abundance of the clusters #312, #243 #188, and #142 relative to CD32a^+^ CD4^+^ T cells for all samples, and heatmap representation showing the phenotype of these clusters. These clusters were significantly less abundant under primary HIV and HIV cART than healthy conditions. In the abundance graph representations, samples from the same HIV-infected patients were represented in the same color. For each condition, the mean cell abundance is indicated (black lines). Statistical differences between conditions were calculated using an unpaired Student’s *t*-test with a *p*-value threshold of 0.05. For heatmap representations, the relative marker expression for each cluster was indicated by a five-tiered color scale ranging from white (not expressed) to dark red (highly expressed).

Five DACs (#5, #258, #136, #86, and #261) were significantly more abundant in HIV^+^ patients in primary infection and after cART than in healthy donors (Figures 3A,B). One, cluster #5, was also significantly (*p* = 0.045) more abundant in primary HIV infection than after cART (Figure 3A). This cluster displayed an activated T_Eff/Mem_ (CCR7^−^ CD45RA^−^ HLA-DR^mid^ CD38^+^) phenotype, expressing the activating immune-receptor LILRA2 and the high-affinity IgG receptor CD64. Cluster #5 was also characterized by the expression of CD57, which is associated with replicative senescence, NKG2C-activating receptor, and the HIV-restriction factor CD317 (also known as BST2 or Tetherin) (Figure 3A).

We also analyzed the phenotype of the four other DACs that showed statistically significant differences in abundance between HIV-infected patients and healthy donors (Figure 3B). Two (#258 and #136) also displayed a T_Eff/Mem_ phenotype (CCR7^−^ CD45RA^−^), expressing LILRA2, CD64, and CX3CR1 (Figure 3B). However, cluster #258 showed an activated phenotype (HLA-DR^+^ CD38^mid^), with co-expression of CD57, NKG2C, CD317, CD161, CD33, and CD11c, whereas cluster #136 displayed a resting profile (HLA-DR^mid^ CD38^−^), with medium expression of PD-L1. Finally, cluster #86 showed a resting T_CM_ phenotype (CCR7^mid^ CD45RA^−^ HLA-DR^mid^ CD38^−^), co-expressing LILRA2, CD64, and CX3CR1, and cluster #261 was associated with an activated T_N_ profile (CCR7^+^ CD45RA^mid^ HLA-DR^mid^ CD38^mid^), co-expressing immunomodulatory receptors LILRA2, CD64, CX3CR1, LILRB4, CD32b, CD161, CD33, costimulatory/inhibitory molecules CD86 and PD-L1, and HIV-restriction factor CD317.

Our analysis demonstrates that specific LILRA2^+^ CD64^+^ CD32a^+^ CD4^+^ T-cell clusters, characterized by naive and memory T-cell phenotypes, were more abundant in HIV^+^ patients from the early phase of infection. Among these clusters, the activated T_Eff/Mem_ CD32a^+^ LILRA2^+^ CD57^+^ NKG2C^+^ cluster (#5) showed a gradient of abundance which was lowest in healthy samples, higher in those undergoing cART, and highest in those with a primary HIV infection. These data suggest that the increase in the proportion of the activated T_Eff/Mem_ CD32a^+^ LILRA2^+^ CD57^+^ NKG2C^+^ subset occurred early during HIV infection and that it remained elevated, but to a lesser extent, after effective cART.

### The Proportion of the LILRA2^−^ CD32a^+^ CD4^+^ T-Cell Subset Is Lower in Primary HIV Infection and cART Stages Than in Healthy Condition

We also investigated whether some clusters of CD32a^+^ CD4^+^ T cells were more abundant in healthy individuals, since a small portion of T cells from healthy donors were previously found to express CD32 (9–11). We thus focused on DACs that were significantly less abundant in primary HIV and cART patients than in healthy donors (Figures 3C,D).

Six DACs (#241, #162, #312, #243, #188, and #142) were significantly less abundant in all HIV^+^ stages than in healthy donors (Figures 3C,D). Clusters #241 and #162 were also significantly less abundant in primary HIV infection than after cART (Figure 3C). These clusters displayed a resting T_Eff/Mem_ phenotype (CCR7^−^ CD45RA^−^ HLA-DR^mid^ CD38^−^) and highly expressed CD161 (Figure 3C). Moreover, they also showed medium expression of CD64, CX3CR1, and CD33 (high for cluster #241). Cluster #241 also displayed medium expression of CD317, CD32b, and CD11c (Figure 3C).

The four other DACs (#312, #243, #188, and #142) showed no statistical difference in abundance between primary HIV-infected patients before and after effective cART (Figure 3D). Two DACs, clusters #312 and #243, were associated with a resting T_CM_ phenotype (CCR7^+^ CD45RA^−^ HLA-DR^mid^ CD38^−^), with medium expression of CD64, CX3CR1, LILRB4, and CD33 for cluster #243 (Figure 3D). Finally, clusters #188 and #142 displayed an activated (HLA-DR^mid^ CD38^+^) and resting (HLA-DR^mid^ CD38^−^) T_N_ profile (CCR7^+^ CD45RA^+/mid^), respectively. They also showed expression of CD64, CX3CR1, and CD33, with cluster #188 co-expressing LILRB4 and PD-L1 and cluster #142 co-expressing CD86, CD161, and CD14.

Our data show that the proportion of CD32a^+^ CD4^+^ T-cell clusters with specific resting memory or resting/activated naive phenotypes in healthy donors are significantly reduced during HIV infection. In contrast to the HIV-associated subsets, these clusters showed low expression of CD64 and no expression of LILRA2. This suggests that LILRA2 expression may be associated with CD32a^+^ CD4^+^ T cells that are preferentially preserved, differentiated, and/or expanded from primary HIV infection to the cART stage. Finally, our data also demonstrate that T_Eff/Mem_ CD32a^+^ LILRA2^−^ CD161^+^ clusters are less represented during primary HIV infection than under cART and healthy conditions, suggesting that early infection induces their depletion or phenotypic modification.

### Cell Abundance of the LILRA2^+^ CD32a^+^ CD4^+^ T-Cell Cluster #5 Correlates With HIV DNA Levels

The total amount of HIV DNA in PBMCs has been shown to correlate with the size of HIV reservoir (30). Therefore, we next investigated whether the abundance of CD32a^+^ CD4^+^ T cells was associated with HIV DNA in PBMCs. There was no correlation between HIV DNA levels and the percentage of total CD32a^+^ cells among CD4^+^ T cells (Figure S7A in Supplementary Material), in accordance with previous studies (10, 11). Thus, we further investigated the association of HIV DNA levels with the abundance of cell populations at the cluster level. Correlations were based on the number of cells associated with each cluster relative to the number of cells in the parent population, corresponding, respectively, to CD32a^+^ CD4^+^ T cells or CD4^+^ T cells.

**Figure 4:**
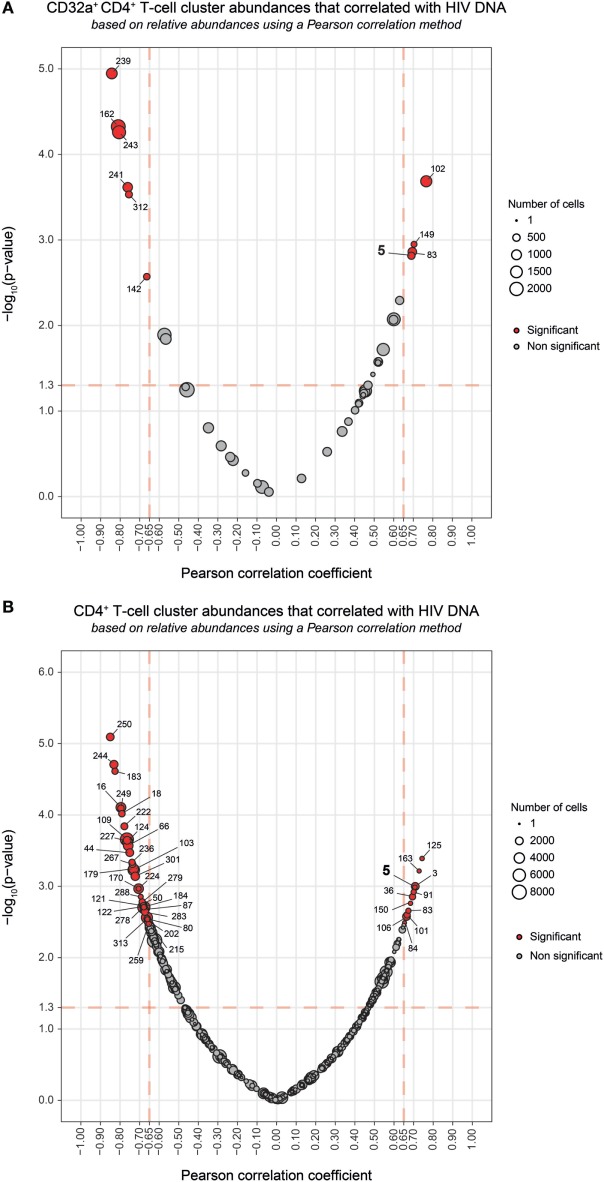
Correlation analyses of CD32a^+^ CD4^+^ and CD4^+^ T-cell cluster abundances with HIV DNA levels. **(A,B)** Correlations were based on the number of cells associated with each cluster relative to the number of cells in the parent population, corresponding to CD32a^+^ CD4^+^ T cells or CD4^+^ T cells, respectively. Clusters having an abundance positively (right) or negatively (left) correlated with total HIV DNA levels are represented in red. The two-dimensional charts represent the association between the cell abundance and total HIV DNA for each cluster using the Pearson correlation coefficient and associated *p*-value. Each dot in the representation corresponds to a cell cluster. Significantly correlated clusters are indicated in red with a Pearson correlation coefficient > 0.65 and a *p*-value < 0.05. The size of the dot is proportional to the number of cells of the whole dataset associated with the cluster. The Pearson correlation coefficient is represented on the *X*-axis and the associated *p*-value, shown as −log10, on the *Y*-axis. Cluster #5 is indicated in bold.

Among the CD32a^+^ CD4^+^ T-cell clusters, 10 significantly (Pearson correlation coefficient > 0.65, *p* < 0.05) correlated with HIV DNA levels (Figure 4A). Six were differentially abundant between HIV^+^ and healthy conditions (Figure 2B). Among them, clusters for which the abundance negatively correlated with HIV DNA levels were those previously identified as resting T_Eff/Mem_ #241 and #162, T_CM_ #312 and #243, and T_N_ #142 clusters. However, these clusters did not negatively correlate with HIV DNA levels among CD4^+^ T-cell clusters (Figure 4B). Our analysis revealed only cluster #5 was differentially abundant between HIV^+^ and healthy conditions and positively correlated with HIV DNA levels (Pearson correlation coefficient = 0.690, *p* = 0.0015) (Figure 4; Figure S7B in Supplementary Material). This correlation was observed among both CD32a^+^ CD4^+^ and CD4^+^ T-cell clusters (Figure 4). Cluster #5 also showed unique significant differences in abundance relative to CD4^+^ T cells between all conditions (Figure S8A in Supplementary Material). This activated T_Eff/Mem_ CD32a^+^ LILRA2^+^ CD57^+^ NKG2C^+^ subset represented an average of 0.37% of primary HIV CD4^+^ T cells vs. only 0.1 and 0.01% under HIV cART and healthy conditions, respectively (Figure S8A in Supplementary Material). We also determined the percentages of cells (relative to CD4^+^ T cells) associated with the clusters described in Figure 3 (Figures S8B–D in Supplementary Material).

We further characterized the phenotype of this activated T_Eff/Mem_ CD32a^+^ LILRA2^+^ CD57^+^ NKG2C^+^ subset by quantifying differences in marker expression between this subset and all CD4^+^ T cells by the Kolmogorov–Smirnov distance (KS) (Figure S9 in Supplementary Material). CD57 showed the highest difference between cluster #5 and the whole set of CD4^+^ T cells (KS = 0.9214). The next most distant markers were CCR7 (KS = 0.6434), CD32a (KS = 0.6387), CD45 (KS = 0.5899), NKG2C (KS = 0.5892), CD38 (KS = 0.4817), HLA-I (KS = 0.4619), CD64 (KS = 0.3763), LILRB1 (KS = 0.3130), and LILRA2 (KS = 0.3036). These results are consistent with the phenotypic characterization of cluster #5 displayed in the heatmap representation (Figure 2A). Moreover, these data provide further details on the specificity of the markers associated with this cluster that could be helpful for cell sorting.

Overall, our results show that the abundance of the LILRA2^+^ CD32a^+^ CD4^+^ T-cell cluster, displaying an activated T_Eff/Mem_ phenotype and co-expressing CD57, NKG2C, and CD317 positively correlated with HIV DNA levels.

## Discussion

A previous study has proposed CD32a as a specific marker of CD4 T latently infected cells (7). However, CD32a was also reported on CD4^+^ T cells showing HIV replication and on CD4^+^ T cells from healthy donors; suggesting a complex relationship between HIV infection and CD32a expression on CD4^+^ T cells (8–11). Here, we aimed to better characterize the heterogeneity and abundance of CD32a^+^ CD4^+^ T cells in various conditions including primary HIV infection, cART, and healthy conditions. Indeed, CD4^+^ T cells include various subsets that are selectively activated during early HIV infection, resulting in changes in protein expression and clonal expansion. In addition, CD4^+^ T-cell subsets are differentially infected and killed by HIV, thus increasing the complexity of their dynamics during the infection.

High-dimensional analyses are critical for disentangling the numerous cellular and molecular events induced by HIV infection. Our mass cytometry panel included typical and atypical T-cell markers that may be associated with the uncommon expression of CD32a on CD4^+^ T cells during HIV infection. Moreover, all previous studies carried out flow-cytometry analysis using a pan anti-CD32 antibody that binds both CD32a and CD32b receptors, possibly leading to misinterpretation of the data (7, 10). We thus included antibodies that make it possible to clearly distinguish CD32a from CD32b isoforms (Figure S1 in Supplementary Material). Hence, our data unambiguously reflect CD4^+^ T cells expressing CD32a, but not CD32b, protein on their surface (Figure 2A).

Our study showed that CD32a^+^ CD4^+^ T cells can be found not only in HIV^+^ patients but also in healthy donors and encompass heterogeneous populations. Indeed, we detected CD32a^+^ CD4^+^ T cells from HIV-infected patients and healthy donors within T_CM_, T_Eff/Mem_, and T_N_ subsets. Under cART and healthy conditions, the proportion of CD32a^+^ T_CM_ was significantly higher than CD32a^+^ T_N_ (Figure S6 in Supplementary Material). These data are in accordance with previous reports showing CD32 expression on small subsets of CD4^+^ or CD8^+^ T cells from healthy donors (9–11). Moreover, we also showed that some CD32a^+^ CD4^+^ T-cell subsets were more abundant under healthy than HIV^+^ conditions (Figures 3C,D). Such subsets of CD32a^+^ CD4^+^ T cells may be more prone to depletion or modification of protein expression during HIV infection, resulting in the negative correlation of their abundance with total HIV-DNA levels observed in our study (Figure 4A).

We analyzed the abundance of CD32a^+^ CD4^+^ T-cell clusters in HIV^+^ patients in the primary-infection phase and after effective cART and compared them with those from healthy donors (Figure 3). CD32a^+^ CD4^+^ T-cell clusters co-expressing LILRA2 and CD64 were highly represented in all HIV^+^ stages, in contrast to healthy conditions. Conversely, the proportion of CD32a^+^ CD4^+^ T-cell clusters with lower CD64 and negative LILRA2 expression was higher under healthy conditions. These data suggest that LILRA2 expression may be associated with CD32a^+^ CD4^+^ T cells that are preserved, differentiated, and/or expand in primary HIV infection and maintained after effective cART. A previous study reported that LILRA2 is expressed by monocytes and neutrophils and recognizes bacterially cleaved immunoglobulin, leading to the activation of signaling pathways and subsequent immune responses (31). Thus, CD64^+^ LILRA2^+^ CD32a^+^ CD4^+^ T-cell populations that increase in proportion during primary HIV infection and cART treatment may be more prone to activation through the engagement of CD32a, CD64, or LILRA2 activating receptors. Given that CD4^+^ T cells can uptake membrane fragments during cell–cell interactions through a process named trogocytosis (32, 33), it is also possible that the expression of uncommon myeloid markers on the surface of CD32a^+^ CD4^+^ T cells could result from intercellular membrane exchanges. Analysis of the distribution of these molecules on CD4^+^ T cells by confocal microscopy but also characterization at mRNA level should be helpful to determine the implication of trogocytosis in the unusual markers found on CD32a^+^ CD4^+^ T cells.

Among LILRA2^+^ CD32a^+^ CD4^+^ T-cell clusters that were more abundant in HIV^+^ stages, only cluster #5 was also more abundant during primary HIV infection than during cART treatment. This cluster showed an Eff/Mem phenotype and displayed high expression of CD38, CD57, NKG2C, and CD317 restriction factor for HIV that retains nascent virions at the cell surface, thus preventing their release (34). Expression of NKG2C was reported on a rare CD4^+^ T-cell population in pathological situations, such as multiple sclerosis or cytomegalovirus (CMV) infection (35, 36). In addition, the expression of CX3CR1 was reported on CMV-specific CD4^+^ T cells in patients showing HIV-associated atherosclerosis (37). In this regard, CMV serological status was assessed in HIV^+^ samples and demonstrated that patients were already positive for IgG (but not IgM), indicating they were not in the primary stage of CMV infection. In addition, no clinical parameters associated with CMV reactivation were detected during our study, ruling-out the implication that cluster abundance modification was a result of CMV activity.

Previous studies based on classical CD4^+^ T-cell markers could not detect any correlation between the percentage of CD32^+^ CD4^+^ T-cell subsets and the amount of HIV DNA (10, 11). We found a single cluster (#5) that positively correlated with HIV DNA levels, not only among the CD32a^+^ CD4^+^ T-cell clusters but also the CD4^+^ T-cell clusters (Figure 4; Figure S7B in Supplementary Material). These results suggest that not all CD32a^+^ CD4^+^ T cells are associated with HIV DNA, but only specific subsets, including the activated T_Eff/Mem_ CD32a^+^ LILRA2^+^ CD57^+^ NKG2C^+^ subset. The abundance of this subset was significantly higher in HIV^+^ samples than those from healthy donors and also higher in primary HIV infection than after cART. It was previously reported that the total amount of HIV DNA reflects the global HIV reservoir (30). Thus, due to its formation during the early stages of HIV infection and its maintenance under cART, this CD32a^+^ LILRA2^+^ CD57^+^ NKG2C^+^ T_Eff/Mem_ cluster displaying an activated profile may contribute to HIV persistence as an active reservoir. However, our analysis does not directly show the presence of higher levels of HIV DNA in CD4^+^ T cells belonging to this cluster. Moreover, total HIV DNA included integrated and non-integrated viral genomes coding for both competent and defective viruses. Therefore, future studies with purified CD32a^+^ CD4^+^ T cells are required to determine if there is an enrichment with HIV DNA within this cluster and if this DNA supports viral transcription.

Our analysis also revealed that numerous CD32a^+^ CD4^+^ T-cell clusters show expression of HLA-DR. These data are in agreement with a recent study demonstrating that a high portion of CD32^+^ CD4^+^ T cells from HIV-infected patients under cART express HLA-DR (38). In this regard, most of the samples analyzed in our study were collected from HIV^+^ patients under cART, or in early phase of HIV infection (before cART), which is associated with enhanced immune activation and high viral load. Hence, it is likely that the samples acquired from HIV^+^ patients may account for the elevated number of HLA-DR^+^ CD32a^+^ CD4^+^ T-cell clusters found in our analysis. Moreover, concordantly with the study from Abdel-Mohsen et al., our results show that some activated, and not resting, CD32a^+^ CD4^+^ T-cell clusters positively correlated with the level of HIV DNA (38).

In conclusion, our results unravel the landscape and abundance modifications of CD32a^+^ CD4^+^ T cells induced from early HIV infection to effective cART. Overall, this study brings a new view of CD32a^+^ CD4^+^ T cells during HIV infection that could be helpful in the discussions about HIV persistence and reservoirs.

## Materials and Methods

### Study Subjects and Ethics Statements

All 12 subjects gave written informed consent to participate in the study. This study involved patients with primary HIV-1 infection enrolled in the French ANRS CO6 PRIMO cohort, which was approved by the Ethics Committee of Cochin Hospital. Patients were enrolled in the study if HIV infection was estimated to have occurred less than 30 days previously. All patients were antiretroviral naive and were negative for hepatitis B and C viruses. Even though the HIV^+^ patients show previous CMV infection (IgM^−^/IgG^+^), there were no clinical signs of reactivation of CMV since HIV diagnosis. Blood samples were collected at enrollment. Patients were then placed under antiretroviral treatment, and 12 months later, new blood samples were collected. The plasma HIV-RNA load was measured, as well as the number of total HIV-DNA copies per million PBMCs. CD4^+^ T cells were also counted for each sample before and after cART. All primary HIV-infected patients showed a viral load of fewer than 50 copies of viral RNA/ml and had more than 500 CD4^+^ T cells/μl of blood once under cART. This study also involved blood samples of healthy subjects obtained from the Etablissement Français du Sang.

### Sample Processing and Storage

PBMCs from each sample were isolated by Ficoll density gradient centrifugation. The number of viable PBMCs was determined using an automated blood counter (Vi-Cell/Beckman Coulter). Approximately 10 million PBMCs were put in a cryotube with 90% heat-inactivated FBS (Eurobio) and 10% DMSO (Sigma). All samples were stored at −150°C in cryotubes.

### HIV DNA Quantification

Total HIV DNA was quantified in PBMCs by qPCR (Biocentric, Bandol, France), as previously described (39).

### Antibodies

Antibodies (listed in Table S1 in Supplementary Material) were either pre-conjugated from the manufacturer (Fluidigm, San Francisco, CA, USA) or conjugated in-house with the appropriate metal isotopes. Between 200 and 400 µg pure mAbs (carrier-protein-free) from various manufacturers were coupled to Lanthanide from the MaxPar Antibody Labeling Kit X8 4Rxn (Fluidigm). Conjugated Abs were adjusted to 1 µg/µl in Ab Stabilizer Solution (Candor, Biosciences, Wangen, Germany), supplemented with 0.01% sodium azide (Santa Cruz Biotechnology), and stored at 4°C. Abs conjugated in-house, as well as those obtained pre-conjugated, were titrated and validated on samples that were processed identically to those used in the study.

### Cell Staining

All samples were stained on the same day with the same batch of antibodies and acquired within 1 day to avoid batch effects and instrument signal fluctuations. Cells were thawed, and 200 µl of the suspension containing 5 × 10^6^ PBMCs transferred per well. PBMCs were incubated 20 min at 37°C with 1 µM Rhodium DNA-intercalator (Fluidigm). Cells were then washed twice with staining buffer (BD Biosciences) and stained with the primary surface antibody mix in a total volume of 60 µl staining buffer. Cells were incubated for 1 h at 4°C and washed twice. The secondary surface antibody mix was added to the cells in a total volume of 50 µl staining buffer and incubated for 15 min at 4°C. After two washes, cells were resuspended in 200 µl 1.6% PFA (EMS 15710) in PBS (Invitrogen) and incubated 20 min at 4°C. Cells were then washed twice in permeabilization buffer (eBioscience) and incubated 30 min at 4°C in 200 µl permeabilization buffer with 1 µM Iridium DNA-intercalator (Fluidigm). After two washes, PBMCs were incubated at 4°C overnight in 200 µl 1.6% PFA with 0.1 µM Iridium DNA-intercalator. For acquisition, cells were washed once with staining buffer, once with PBS, and twice with ddH_2_O and filtered through a cell strainer cap of a 5-ml polystyrene round-bottom tube (BD Biosciences). Fifty microliters of normalization beads (Fluidigm) were added to each sample. Then, samples were acquired using a mass cytometer (CyTOF; Fluidigm) following the standard procedure recommended by the manufacturer. An average of 207,233 ± 17,141 events was acquired per sample.

### Data Normalization

Raw cytometry profiles generated by CyTOF were normalized with the MatLab Compiler software normalizer (40), using the signal from the normalization beads, as recommended by the software developers.

### Manual Gating of CD45^+^ CD4^+^ T Cells

Normalized events were gated to exclude doublets and triplets from the analysis by selecting cells with only one Iridium level. Then, beads added for normalization and dead cells were removed by selecting cells negative for Cerium (Ce140) and Rhodium, respectively. Finally, leukocytes were selected based on the positive expression of CD45. CD4^+^ T cells were then selected based on the expression of CD3, CD19, CD4, and CD8 (Figure S2 in Supplementary Material). After this gating strategy, an average of 28,305 ± 3,375 cells remained per sample.

### Automatic Identification of Cell Clusters

The automatic identification of cell clusters was performed using SPADE with the publicly available R package. We observed large differences in the number of manually gated CD4^+^ T cells in each sample, ranging from 6,864 to 53,599 cells. We avoided over-representation of cell populations present in samples with a high number of cells by first randomly selecting 6,864 cells for each sample. This number corresponded to the sample with the lowest number of cells. These uniformly down-sampled events were up-sampled at the end of the SPADE analysis to better ascertain the phenotype of identified clusters. The SPADE heatmap was generated using 32 clustering markers (shown in blue Figure 1B). CD8 and CD19 were discarded from the SPADE analysis as we used them to pre-select CD4^+^ T cells and CX3CR1 was not included in the set of clustering markers due to high heterogeneity of expression among samples. SPADE was parametrized to identify 350 cell clusters using a down-sampling parameter of 40%. These parameters were found to be the most efficient to obtain clusters with uniform phenotypes with a sufficient number of associated cells by SPADEVizR (29). Uniform clusters were defined as those with unimodal density expression (Hartigan’s dip test) and a low range of expression (IQR < 2) for all clustering markers.

### Phenotypic Characterization of Identified Cell Clusters

The phenotypic categorization of the 350 cell clusters was performed using SPADEVizR (29), based on the average median expression of each marker for all individuals. Marker expression was classified into five relative expression categories. The categories were defined based on the range of expression (5th to 95th percentile) of each marker relative to that of CD4^+^ T cells. The marker intensity ranges were then divided into five uniform categories representing negative, low, medium, high, and very high marker expression and represented using a color scale ranging from white to dark red on the heatmaps. Hierarchical clustering was computed based on the Euclidian distance, using the complete linkage method.

Clusters displaying a myeloid phenotype were identified among the 350 cell clusters and removed from the SPADE analysis, leaving only CD4^+^ T-cell clusters. In addition, clusters with less than 40 cells were also removed from the analysis, as their phenotypes could not be assessed accurately. Thus, 322 CD4^+^ T-cell clusters remained from the 350 SPADE clusters. Finally, CD4^+^ T-cell clusters displaying high or very high expression of CD32a (named CD32a^+^) were selected to perform all analyses.

Detailed phenotypic characterization of cluster five relative to other CD4^+^ T-cell clusters was performed using CytoCompare (41), based on the KS, using a threshold of 0.30.

### Identification of DACs and Correlating Clusters

Differentially abundant clusters were identified using SPADEVizR (29), based on the percentage of cells in the clusters relative to total CD32a^+^ CD4^+^ T cells by unpaired Student’s *t*-tests with a fold-change threshold of 2 and a *p*-value threshold of 0.05. Correlated clusters (CCs) were identified based on the percentage of cells in clusters relative to either CD32a^+^ CD4^+^ or CD4^+^ T cells that correlated with total HIV DNA levels. CCs were also identified using SPADEVizR and based on the Pearson correlation coefficient with a threshold of 0.65 and a *p*-value < 0.05.

### Data Availability

Normalized cytometry profiles are available on the FlowRepository database through accession number FR-FCM-ZYGJ.

## Ethics Statement

All 12 subjects gave written informed consent to participate in the study. This study involved patients with primary HIV-1 infection enrolled in the French ANRS CO6 PRIMO cohort, which was approved by the Ethics Committee of Cochin Hospital.

## Author Contributions

Conceptualization: SC, A-SB, OL, RG, and BF. Methodology: SC, NT, LA, A-SB, OL, RG, and BF. Validation: SC, NT, LA, BV, CB, CG, VA-F, CL, PB, RG, A-SB, OL, and BF. Formal analysis: SC, NT, LA, VA-F, CB, CG, BV, A-SB, OL, RG, and BF. Investigation: SC, LA, NT, and BF. Resources: CG, VA-F, CL, PB, and A-SB. Writing—original draft: SC, NT, and BF. Writing—review and editing: SC, NT, BV, CB, CG, VA-F, PB, RG, A-SB, OL, and BF. Supervision: RG, A-SB, OL, and BF.

## Conflict of Interest Statement

The authors declare that the research was conducted in the absence of any commercial or financial relationships that could be construed as a potential conflict of interest.

## Supplementary Material

Figure S1 Characterization of CD32a and CD32b antibody specificity by mass cytometry. Representative analysis of metal-conjugated CD32a-Dy161 (upper panels) and CD32b-Sm149 (lower panels) antibody staining of monocytes, B cells, and CD4^+^ T cells performed on PBMCs from one healthy donor (out of six) using FlowJo software.

Figure S2 Gating strategy used to identify CD4^+^ T cells. “Singlets” were identified using cell length vs. Ir191-DNA intercalator and calibration beads were excluded (cells no beads). Living leukocytes were identified by selecting Rhodium (Rh103)Di-negative cells and then CD45^+^ cells. Finally, CD4^+^ T cells were identified by gating on CD3^+^ CD19^−^ and then CD4^+^ CD8^−^ cells.

Figure S3 Phenotypic landscape of CD4^+^ T-cell Spanning-tree Progression Analysis of Density-normalized Events (SPADE) clusters. A heatmap showing relative marker expression for SPADE clusters was generated. The mean of the median expression of each marker was determined and classified in a five-tiered color scale, from white (not expressed) to dark red (highly expressed), according to their range of expression (5th to 95th percentile) throughout the dataset. Clustering markers are shown in blue. Hierarchical clustering of both the cell clusters and clustering markers were performed and are represented by dendrograms.

Figure S4 Relative range of marker expression of Spanning-tree Progression Analysis of Density-normalized Events clusters. Graph showing the relative range of marker expression of clusters obtained after manual gating of CD4^+^ T cells. The range of expression for each marker (5th to 95th percentiles of expression throughout the dataset) are represented using a five-tiered color scale ranging from white (not expressed) to dark red (highly expressed). Clustering markers are shown in blue.

Figure S5 Cell number in each CD32a^+^ CD4^+^ T-cell cluster. This representation shows the number of cells associated with each CD32a^+^ CD4^+^ T-cell cluster, regardless of sample cell origin. Cluster names are indicated on the *X*-axis and the corresponding number of cells on the *Y*-axis. The size of the dots is proportional to the number of cells in the cluster.

Figure S6 Percentages of CD32a^+^ CD4^+^ T_N_, T_CM_, and T_Eff/Mem_ subsets among CD4^+^ T cells from HIV-infected patients and healthy donors. This representation shows the percentage of naive (T_N_), central memory (T_CM_), and effector/memory (T_Eff/Mem_) CD4^+^ T cells among CD32a^+^ CD4^+^ T cells for primary HIV-infected patients before (primary HIV, red circles) and after 12 months of combination antiretroviral treatment (HIV cART, blue squares) and that of healthy donors (healthy, green triangles).

Figure S7 Correlation analysis of total CD32a^+^ CD4^+^ T-cell cluster and cluster #5 cell abundances with HIV DNA levels. **(A)** Correlation analysis of total CD32a^+^ CD4^+^ T-cell cluster cell abundances with total HIV DNA levels. The HIV DNA load (log_10_ copies/10^6^ PBMCs) for each sample are indicated on the *X*-axis, and the associated percentage of cells relative to CD4^+^ T cells for CD32a^+^ CD4^+^ T-cell clusters on the *Y*-axis. The Pearson correlation coefficient was equal to 0.4329 (*p* = 0.0727). **(B)** Correlation analysis between cluster #5 cell abundance and HIV DNA levels. For each sample, the HIV DNA load (log_10_ copies/10^6^ PBMCs) is indicated on the *X*-axis, and associated percentage of cells relative to CD32a^+^ CD4^+^ T cells for cluster #5 on the *Y*-axis. The Pearson correlation coefficient was equal to 0.6902 (*p* = 0.0015).

Figure S8 Percentages of cells associated with clusters #5, #258, #136, #86, #261, #241, #162, #312, #243, #188, and #142 among CD4^+^ T cells in HIV-infected patients and healthy donors. **(A–D)** Graphs showing the cell abundance relative to that of CD4^+^ T cells for clusters previously shown to be differentially abundant between HIV-infected patients (primary HIV and HIV cART) and healthy donors (healthy) among CD32a^+^ CD4^+^ T cells. **(A,B)** Clusters #5, #258, #136, #86, and #261 were significantly more abundant in Primary HIV (circle) and HIV cART (square) than Healthy (triangle) samples among CD32a^+^ CD4^+^ T cells. Cluster #5 was also more abundant in primary HIV than in HIV cART samples among CD32a^+^ CD4^+^ T cells. **(C,D)** Clusters #241, #162, #312, #243, #188, and #142 were significantly less abundant in primary HIV (circle) and HIV cART (square) than in healthy (triangle) samples among CD32a^+^ CD4^+^ T cells. Clusters #241 and #162 were also less abundant in primary HIV than in HIV cART samples among CD32a^+^ CD4^+^ T cells. Samples from the same HIV-infected patients are shown in the same color. For each condition, the mean cell abundance is indicated (black lines). Statistical differences between conditions were calculated using an unpaired Student’s *t*-test with a *p*-value threshold of 0.05.

Figure S9 Marker expression densities showing the phenotypic specificity of cluster #5 relative to whole CD4^+^ T-cell clusters. The expression densities of all clustering markers and CX3CR1 are shown for cluster #5 and whole CD4^+^ T-cell clusters. The marker expression densities for cluster #5 are shown in blue whereas the marker expression densities for all CD4^+^ T-cell clusters are shown in gray. The differences between marker expression densities were quantified using the Kolmogorov–Smirnov distances (KS).

Table S1 Overview of the mass cytometry panel. The metal, antigen, clone, isotype, and supplier are indicated for each antibody. Non-clustering markers are indicated by a star.
