# Supplementary figures and images for: Mass Cytometry Analysis Reveals the Landscape and Dynamics of CD32a+ CD4+ T Cells From Early HIV Infection to Effective cART
Source: Front Immunol. 2018 Jun 4;9:1217. doi: 10.3389/fimmu.2018.01217 (PMC5995043; doi:10.3389/fimmu.2018.01217)

A

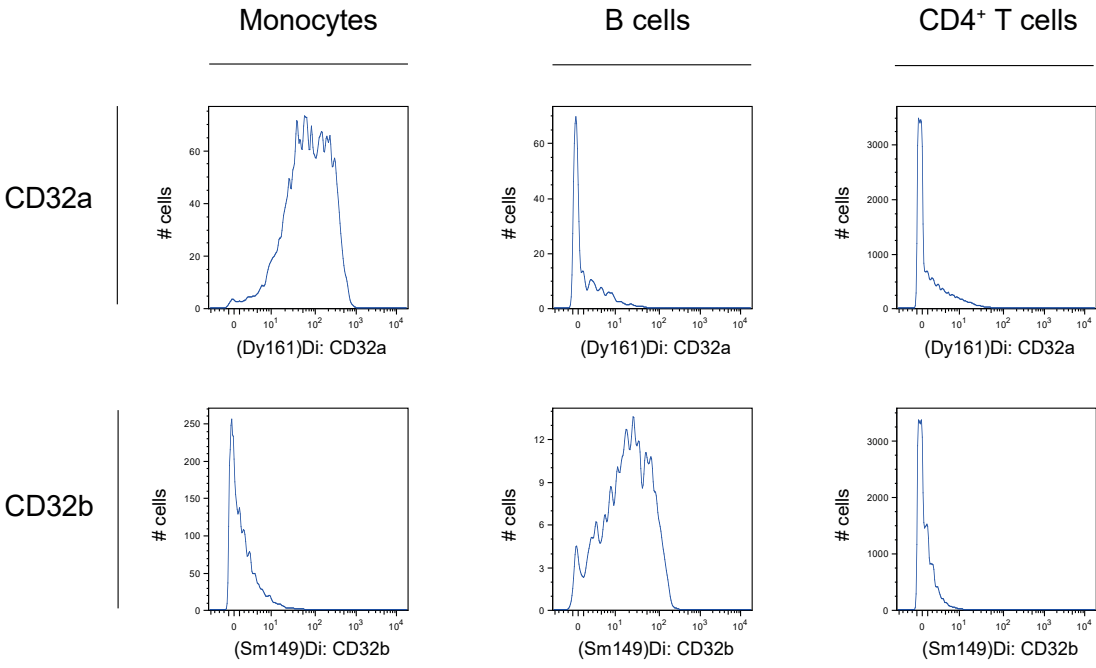

Supplement: Figure S1 — Characterization of CD32a and CD32b antibody specificity by mass cytometry. Representative analysis of metal-conjugated CD32a-Dy161 (upper panels) and CD32b-Sm149 (lower panels) antibody staining of monocytes, B cells, and CD4+ T cells performed on PBMCs from one healthy donor (out of six) using FlowJo software. [file image_1.PDF]

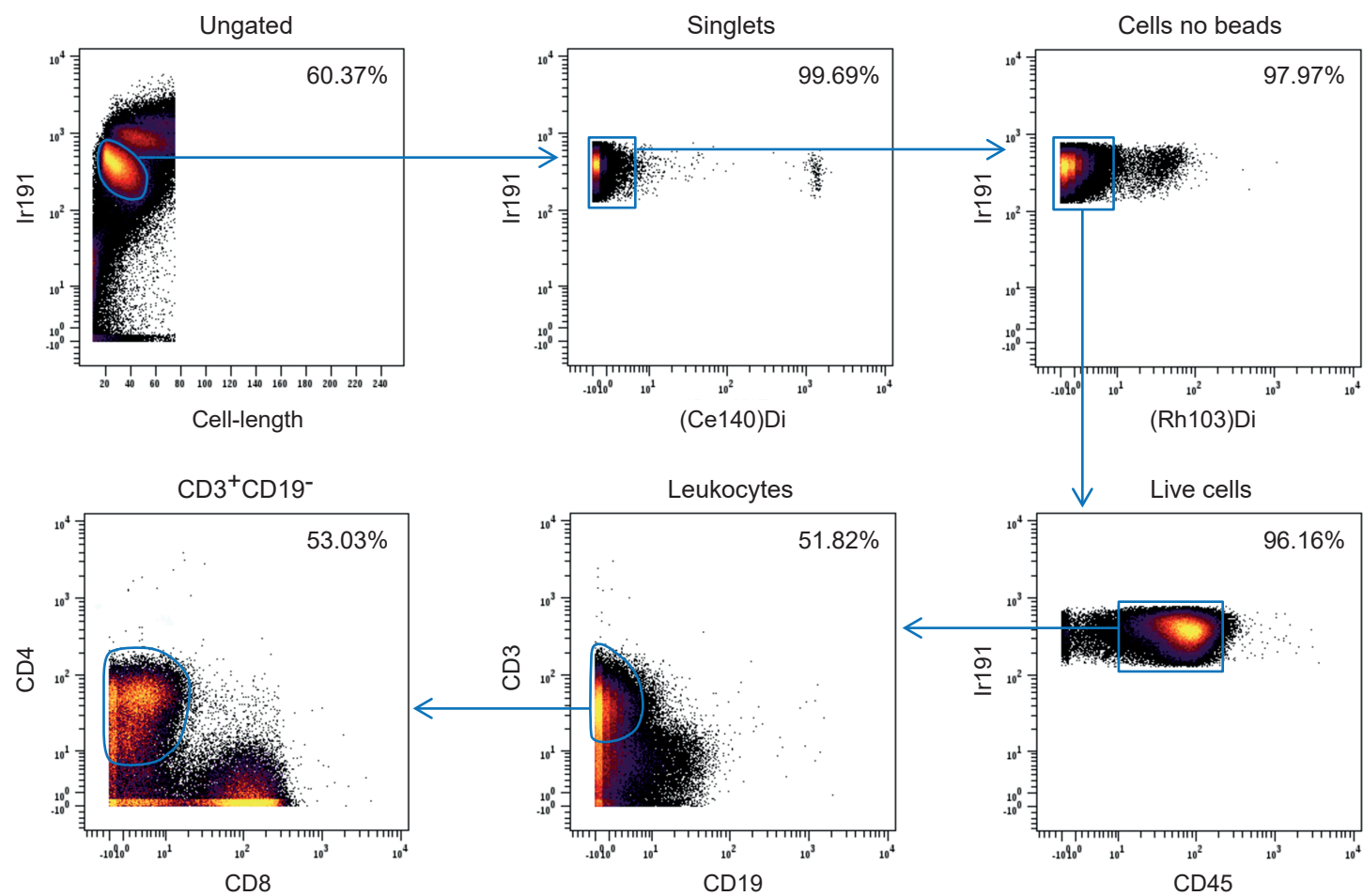

Supplement: Figure S2 — Gating strategy used to identify CD4+ T cells. “Singlets” were identified using cell length vs. Ir191-DNA intercalator and calibration beads were excluded (cells no beads). Living leukocytes were identified by selecting Rhodium (Rh103)Di-negative cells and then CD45+ cells. Finally, CD4+ T cells were identified by gating on CD3+ CD19− and then CD4+ CD8− cells. [file image_2.PDF]

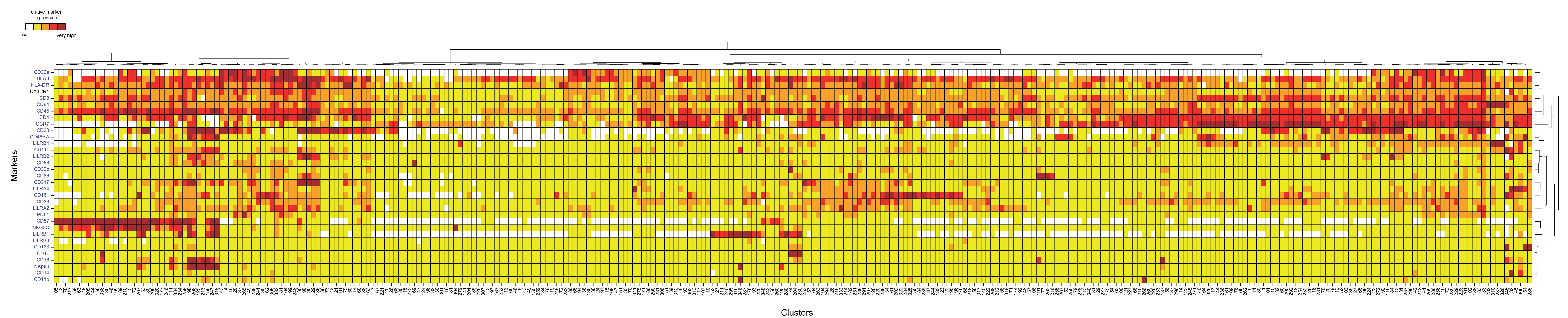

Supplement: Figure S3 — Phenotypic landscape of CD4+ T-cell Spanning-tree Progression Analysis of Density-normalized Events (SPADE) clusters. A heatmap showing relative marker expression for SPADE clusters was generated. The mean of the median expression of each marker was determined and classified in a five-tiered color scale, from white (not expressed) to dark red (highly expressed), according to their range of expression (5th to 95th percentile) throughout the dataset. Clustering markers are shown in blue. Hierarchical clustering of both the cell clusters and clustering markers were performed and are represented by dendrograms. [file image_3.PDF]

Number of cells for each CD32a<sup>+</sup> CD4<sup>+</sup> T-cell cluster

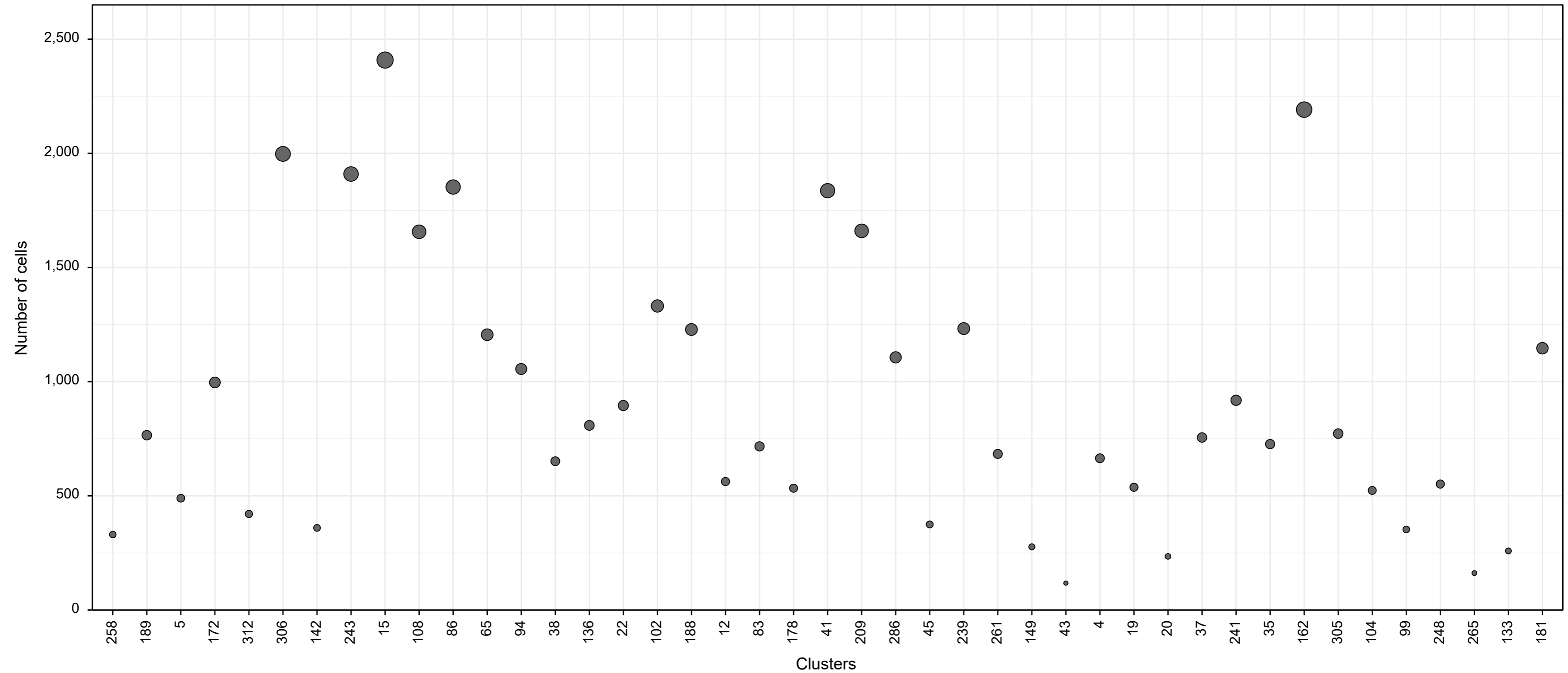

Supplement: Figure S5 — Cell number in each CD32a+ CD4+ T-cell cluster. This representation shows the number of cells associated with each CD32a+ CD4+ T-cell cluster, regardless of sample cell origin. Cluster names are indicated on the X-axis and the corresponding number of cells on the Y-axis. The size of the dots is proportional to the number of cells in the cluster. [file image_5.PDF]

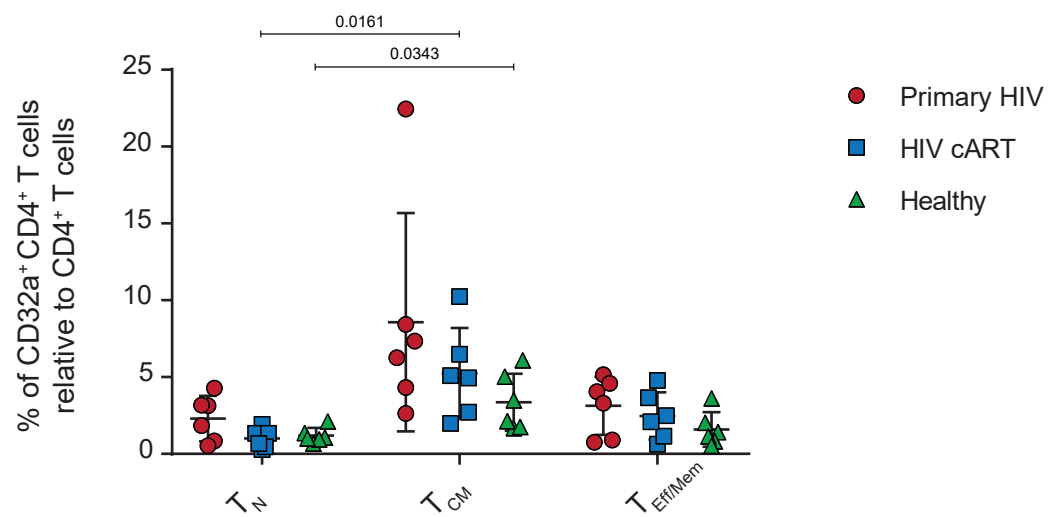

Supplement: Figure S6 — Percentages of CD32a+ CD4+ TN, TCM, and TEff/Mem subsets among CD4+ T cells from HIV-infected patients and healthy donors. This representation shows the percentage of naive (TN), central memory (TCM), and effector/memory (TEff/Mem) CD4+ T cells among CD32a+ CD4+ T cells for primary HIV-infected patients before (primary HIV, red circles) and after 12 months of combination antiretroviral treatment (HIV cART, blue squares) and that of healthy donors (healthy, green triangles). [file image_6.PDF]

A

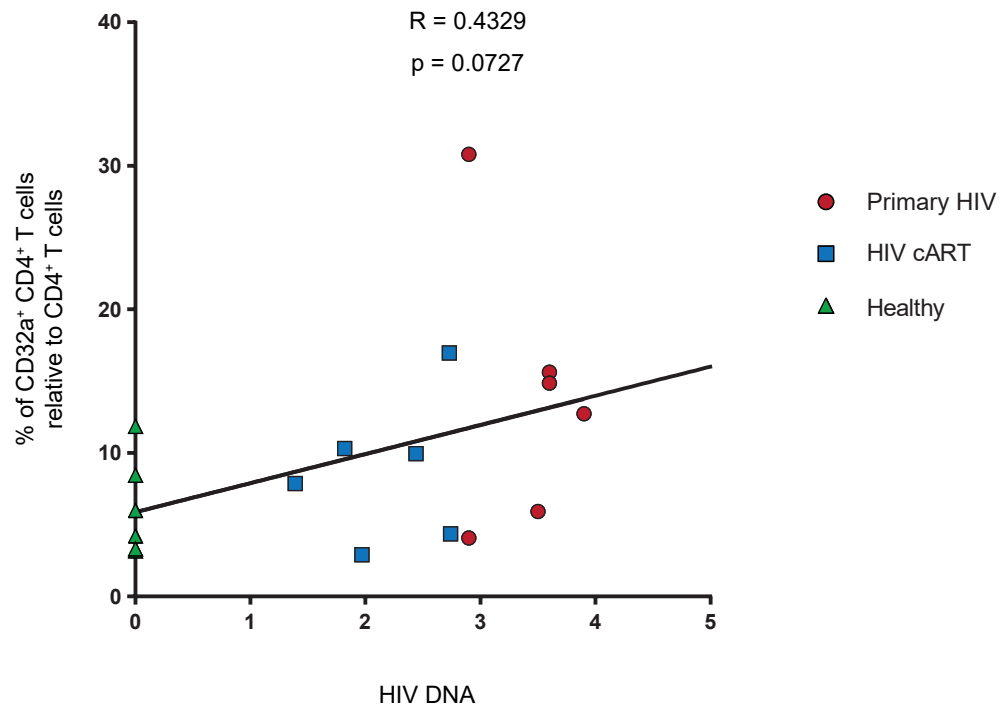

B

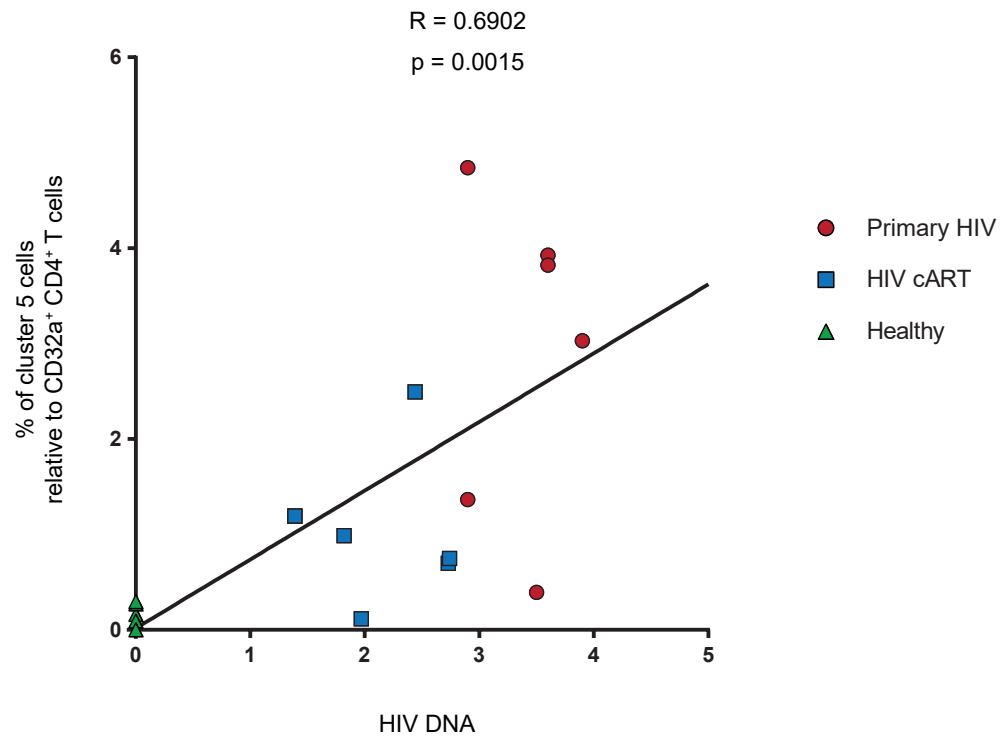

Supplement: Figure S7 — Correlation analysis of total CD32a+ CD4+ T-cell cluster and cluster #5 cell abundances with HIV DNA levels. (A) Correlation analysis of total CD32a+ CD4+ T-cell cluster cell abundances with total HIV DNA levels. The HIV DNA load (log10 copies/106 PBMCs) for each sample are indicated on the X-axis, and the associated percentage of cells relative to CD4+ T cells for CD32a+ CD4+ T-cell clusters on the Y-axis. The Pearson correlation coefficient was equal to 0.4329 (p = 0.0727). (B) Correlation analysis between cluster #5 cell abundance and HIV DNA levels. For each sample, the HIV DNA load (log10 copies/106 PBMCs) is indicated on the X-axis, and associated percentage of cells relative to CD32a+ CD4+ T cells for cluster #5 on the Y-axis. The Pearson correlation coefficient was equal to 0.6902 (p = 0.0015). [file image_7.PDF]

A

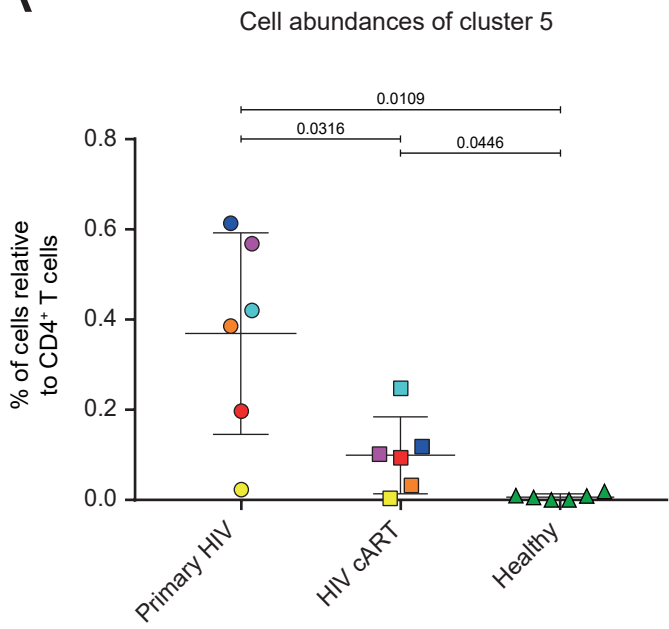

B

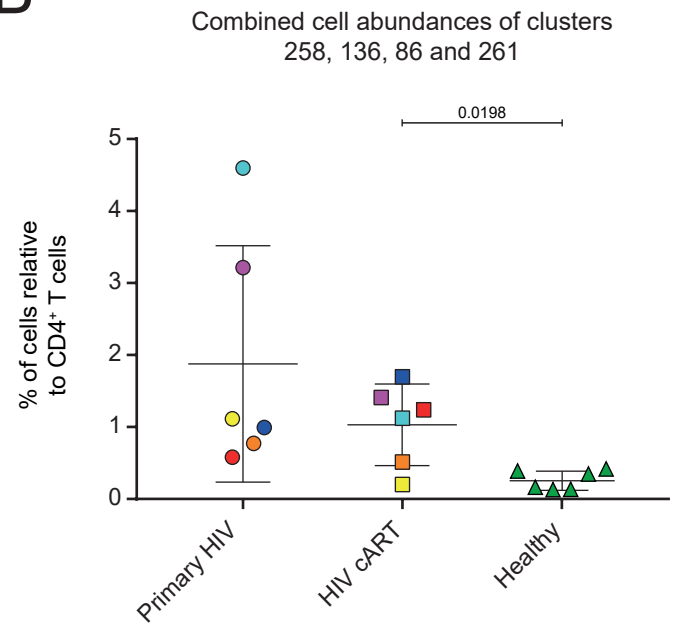

C

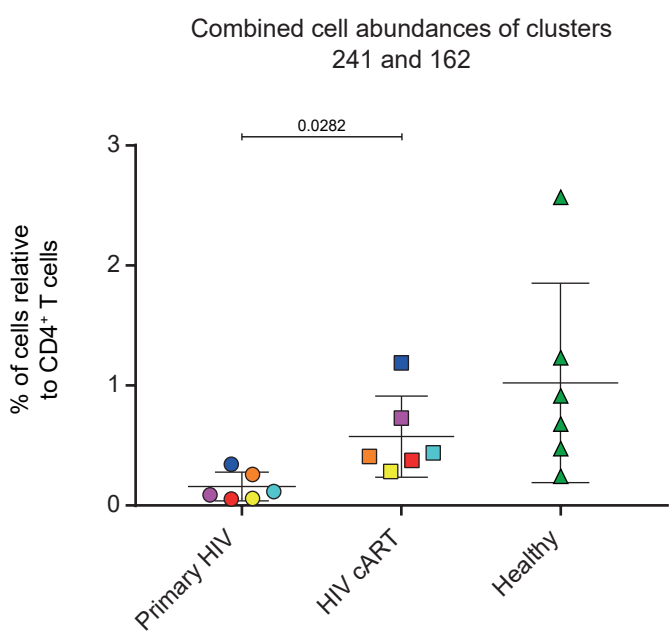

D

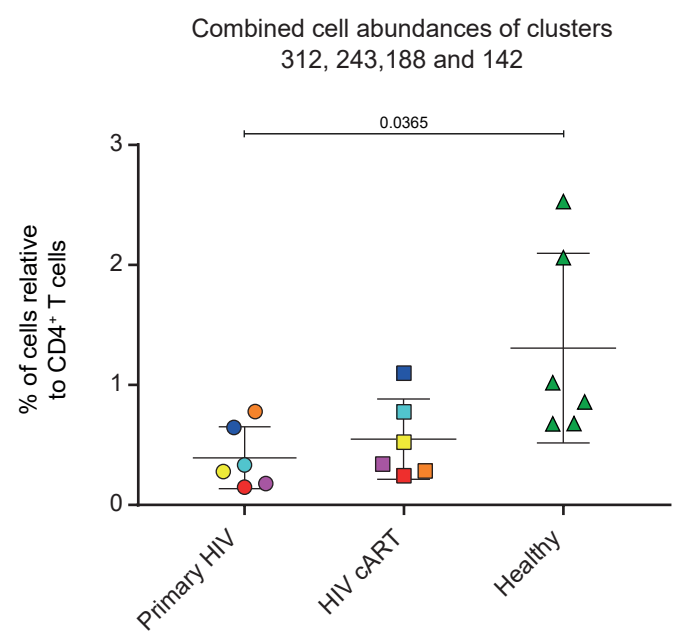

Supplement: Figure S8 — Percentages of cells associated with clusters #5, #258, #136, #86, #261, #241, #162, #312, #243, #188, and #142 among CD4+ T cells in HIV-infected patients and healthy donors. (A–D) Graphs showing the cell abundance relative to that of CD4+ T cells for clusters previously shown to be differentially abundant between HIV-infected patients (primary HIV and HIV cART) and healthy donors (healthy) among CD32a+ CD4+ T cells. (A,B) Clusters #5, #258, #136, #86, and #261 were significantly more abundant in Primary HIV (circle) and HIV cART (square) than Healthy (triangle) samples among CD32a+ CD4+ T cells. Cluster #5 was also more abundant in primary HIV than in HIV cART samples among CD32a+ CD4+ T cells. (C,D) Clusters #241, #162, #312, #243, #188, and #142 were significantly less abundant in primary HIV (circle) and HIV cART (square) than in healthy (triangle) samples among CD32a+ CD4+ T cells. Clusters #241 and #162 were also less abundant in primary HIV than in HIV cART samples among CD32a+ CD4+ T cells. Samples from the same HIV-infected patients are shown in the same color. For each condition, the mean cell abundance is indicated (black lines). Statistical differences between conditions were calculated using an unpaired Student’s t-test with a p-value threshold of 0.05. [file image_8.PDF]

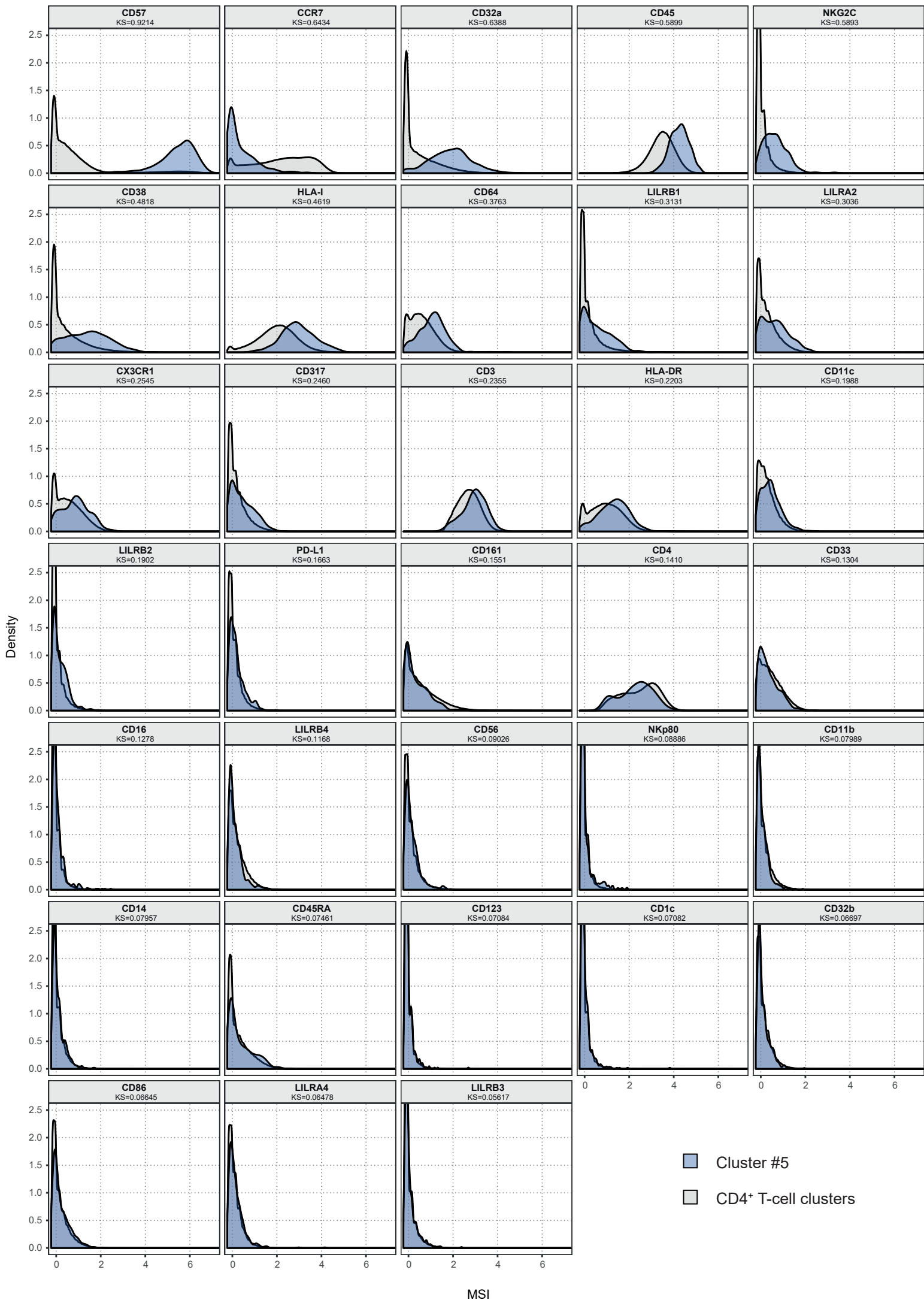

Supplement: Figure S9 — Marker expression densities showing the phenotypic specificity of cluster #5 relative to whole CD4+ T-cell clusters. The expression densities of all clustering markers and CX3CR1 are shown for cluster #5 and whole CD4+ T-cell clusters. The marker expression densities for cluster #5 are shown in blue whereas the marker expression densities for all CD4+ T-cell clusters are shown in gray. The differences between marker expression densities were quantified using the Kolmogorov–Smirnov distances (KS). [file image_9.PDF]
