# Supplementary material for: Mass Cytometry Analysis Reveals the Landscape and Dynamics of CD32a+ CD4+ T Cells From Early HIV Infection to Effective cART
Source: Front Immunol. 2018 Jun 4;9:1217. doi: 10.3389/fimmu.2018.01217 (PMC5995043; doi:10.3389/fimmu.2018.01217)
Supplement: Figure S4 — Relative range of marker expression of Spanning-tree Progression Analysis of Density-normalized Events clusters. Graph showing the relative range of marker expression of clusters obtained after manual gating of CD4+ T cells. The range of expression for each marker (5th to 95th percentiles of expression throughout the dataset) are represented using a five-tiered color scale ranging from white (not expressed) to dark red (highly expressed). Clustering markers are shown in blue. [file image_4.PDF]

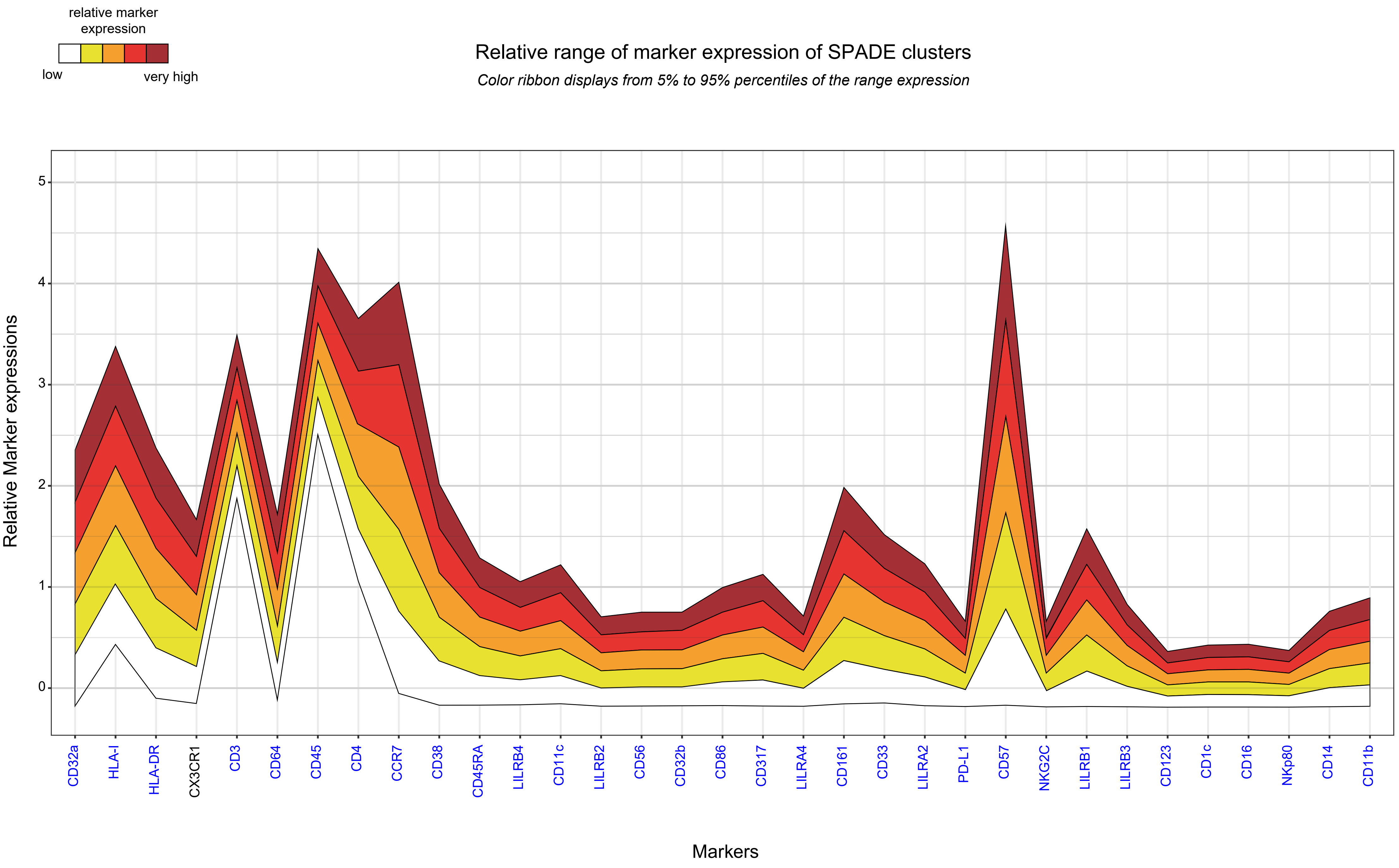
relative marker expression
low
very high
Relative range of marker expression of SPADE clusters
Color ribbon displays from 5% to 95% percentiles of the range expression
Relative Marker expressions
0
1
2
3
4
5
Markers
CD32a
HLA-I
HLA-DR
CX3CR1
CD3
CD64
CD45
CD4
CCR7
CD38
CD45RA
LILRB4
CD11c
LILRB2
CD56
CD32b
CD86
CD317
LILRA4
CD161
CD33
LILRA2
PD-L1
CD57
NKG2C
LILRB1
LILRB3
CD123
CD1c
CD16
NKp80
CD14
CD11b
