# Supplementary material for: Mass Cytometry Analysis Reveals the Landscape and Dynamics of CD32a+ CD4+ T Cells From Early HIV Infection to Effective cART
Source: Front Immunol. 2018 Jun 4;9:1217. doi: 10.3389/fimmu.2018.01217 (PMC5995043; doi:10.3389/fimmu.2018.01217)
Supplement: Table S1 — Overview of the mass cytometry panel. The metal, antigen, clone, isotype, and supplier are indicated for each antibody. Non-clustering markers are indicated by a star. [file table_1.PDF]

Metal	Antigen	Clone	Isotype	Supplier
141	CD11c	3.9	Mouse IgG1, κ	Biolegend
142	HLA-DR	G46-6	Mouse IgG2a, κ	BD BioSciences
143	CD3	UCHT1	Mouse IgG1, κ	BD BioSciences
144	CD64	10.1.1	Mouse IgG1, κ	Miltenyi
145	CD8*	RPA-T8	Mouse IgG1, κ	BD BioSciences
146	CD45	HI30	Mouse IgG1, κ	BD BioSciences
147	CD57	TB03	Mouse IgM, κ	Miltenyi
148	HLA-I	W6/32	Mouse IgG2a, κ	Biolegend
149	CD32b	2B6	Recombinant Human IgG1	Pasteur Institute
150	CD11b	ICRF44	Mouse IgG1, κ	BD BioSciences
151	CD38	AT1	Mouse IgG1	StemCell
152	CD16	B73.1	Mouse IgG1, κ	BD BioSciences
153	LILRB3 (ILT5)	REA207	Recombinant Human IgG1	Miltenyi
154	CD86	2331(FUN)	Mouse IgG1, κ	BD BioSciences
155	CD45RA	5H9	Mouse IgG1, κ	BD BioSciences
156	CD123	7G3	Mouse IgG2a, κ	BD BioSciences
158	CD161	DX12	Mouse IgG1, κ	BD BioSciences
159	CCR7	G043H7	Mouse IgG2a, κ	Biolegend
160	LILRB4 (ILT3)	REA141	Recombinant Human IgG1	Miltenyi
161	CD32a	IV.3	Mouse IgG2b	StemCell
162	CX3CR1*	2A9-1	Rat IgG2b, κ	Biolegend
163	NKG2C	REA205	Recombinant Human IgG1	Miltenyi
164	LILRA2 (ILT1)	REA219	Recombinant Human IgG1	Miltenyi
165	Biotin	1D4-C5	Mouse IgG2a	DVS Sciences
166	LILRB1 (ILT2)	GHI/75	Mouse IgG2b, κ	Miltenyi
167	NKp80	4A4D10	Mouse IgG1, κ	Miltenyi
168	LILRB2 (ILT4)	REA184	Recombinant Human IgG1	Miltenyi
169	CD317	REA202	Recombinant Human IgG1	Miltenyi
170	CD14	M5E2	Mouse IgG2a, κ	BD BioSciences
171	LILRA4 (ILT7)	REA100	Recombinant Human IgG1	Miltenyi
172	CD4	L200	Mouse IgG1, κ	BD BioSciences
173	CD33	AC104.3E3	Mouse IgG1, κ	Miltenyi
174	CD19*	J3.119	Mouse IgG1	Beckman Coulter
175	PD-L1	29E.2A3	Mouse IgG2b, κ	DVS Sciences
176	CD56	HCD56	Mouse IgG1	DVS Sciences
Biotin	CD1c	L161	Mouse IgG1, κ	Biolegend
